# Access to Healthcare for Minors: An Ethical Analysis of Judgments of the European Court of Human Rights

**DOI:** 10.3390/healthcare9101361

**Published:** 2021-10-13

**Authors:** Fabian-Alexander Tietze, Marcin Orzechowski, Marianne Nowak, Florian Steger

**Affiliations:** Institute of the History, Philosophy and Ethics of Medicine, Ulm University, Parkstraße 11, D-89073 Ulm, Germany; marcin.orzechowski@uni-ulm.de (M.O.); marianne.nowak@uni-ulm.de (M.N.); florian.steger@uni-ulm.de (F.S.)

**Keywords:** minors, medical ethics, access to healthcare services, international law, vulnerable population, barriers

## Abstract

The right to non-discriminatory access to healthcare is anchored in the European Convention on Human Rights and other international treaties or guidelines. Since its ratification, the European Convention on Human Rights was made binding in all Member States of the Council of Europe and is interpreted by the European Court of Human Rights (ECtHR). Despite its legal recognition, discrimination in healthcare provision has repeatedly been an issue of medicoethical and political consideration. In this context, minors are particularly in danger of being deprived of this fundamental right. The aim of this study is to analyze the current state of the ECtHR jurisdiction on challenges in accessing healthcare for minor patients. We conducted a systematic search of judgments by the ECtHR using the keywords “healthcare” and “child”. We performed descriptive statistics and qualitative thematic analysis. Our search resulted in *n* = 66 judgments, which were all screened. Access to healthcare for minors played a role in *n* = 21 judgments, which involved applications against *n* = 13 countries. We formed five, partially overlapping categories, which represent recurring themes regarding the research topic. These themes are governance failure (*n* = 11), the status of refugee, asylum seeker or migrant (*n* = 5), parental home (*n* = 5), maternity and birth (*n* = 4) and others (*n* = 2). The normative framework of the ECtHR’s jurisprudence illustrates intersections between social, legal and medicoethical aspects of minors’ discrimination in the healthcare system. It emphasizes the particular vulnerability of children, who require exceptional protection. Inadequate access to healthcare manifests itself in specific situations, such as in the context of migration or staying in public institutions. Healthcare professionals need to be sensitized for such discrimination mechanisms, as they are often at the forefront of encountering structural discrimination in the healthcare system.

## 1. Introduction

Guaranteeing non-discriminatory access to healthcare is a principal duty of every European Member State and anchored in European and international conventions like the Charter of Fundamental Rights of the European Union [1] or The Constitution of the World Health Organization [2]. From a medicoethical perspective, the discriminatory provision of health services touches the principle of justice in the healthcare system, which requires the equal treatment of every patient. Access to healthcare thereby refers to a multidimensional concept with at least four sub-aspects: (1) service availability, (2) utilization of services and barriers to access, (3) relevance and effectiveness of treatment and (4) equity of healthcare provision [3].

Belonging to social minorities has often been discussed as a relevant obstacle in access to health services. The vulnerability concept of social or clinical groups implies a susceptibility to harm or personal threat in the health system [4]. Minors can be especially susceptible to discrimination, because they have limited awareness of healthcare availabilities [5]. Moreover, healthcare professionals often lack an understanding of minors’ ability for decision-making [6]. As a result of their vulnerable condition, minors can experience depersonalizing or paternalistic treatment by healthcare professionals [7]. Additional social and clinical factors, such as differing ethnical origins [5,8], the context of flight [9], minority religious beliefs [10], sexual orientations [11] and complex medical states [7,12] can further worsen the possibility to equally access healthcare. Formation of vulnerability in minor patients is also related to social determinants. These include adverse effects in childhood such as drug addictions or sexual abuse in the parental homes, poverty, access barriers resulting from living in rural areas or physical and mental disabilities [5].

### 1.1. The European Court of Human Rights (ECtHR)

The ECtHR constitutes the highest institution of European jurisdiction interpreting the European Convention on Human Rights (hereafter “the Convention”). The Convention is an international binding agreement guaranteeing fundamental human rights and freedoms in 47 Member States of the Council of Europe. As foreseen by Article 19 of the Convention, the ECtHR is a supranational court interpreting the Convention and judging on human rights violations in all Member States. Decisions of the ECtHR can be only sought if national remedies have been exhausted (Article 35 § 1 of the Convention). In all ratifying countries, its judgments are binding and, if necessary, should lead to the legal amendment. Jurisdiction of the ECtHR also concerns guarantees of healthcare provision, putting the issue of non-discriminatory access to it directly into the context of human rights [13,14] (p. 85). However, the ECtHR often refers in its judgments to other international guidelines and conventions, if the subject matter of a case is only implicitly touched in the Convention. In our case, the Convention on the Rights of the Child (CRC) is an important reference for the ECtHR, as it explicitly codifies the right of minor patients in the context of access to healthcare.

### 1.2. Aims

The aim of our study was to analyze the current state of the ECtHR jurisdiction on challenges in accessing healthcare for minor patients. Analysis of the normative framework of the jurisdiction can illustrate intersections between social, legal and medicoethical aspects of the issue of discrimination in the healthcare system. Our research interest was directed towards discriminative structures and actions, which affect minor patients. Gaining knowledge on this topic can contribute to the successive reduction of barriers in access to healthcare. To our knowledge, this is the first study to provide results from quantitative and qualitative analysis of ECtHR judgments involving the question of access to healthcare for minors. Our analysis was guided by the following research questions: (1) How often did the ECtHR judge on the specific situation of minors regarding access to healthcare? (2) Did judgments of the ECtHR adequately mirror ethical challenges related to barriers in accessing healthcare? (3) How can these judgments be categorized? (4) How can these thematic categories contribute to a better understanding of discrimination in access to healthcare?

## 2. Materials and Methods

**Figure 1:**
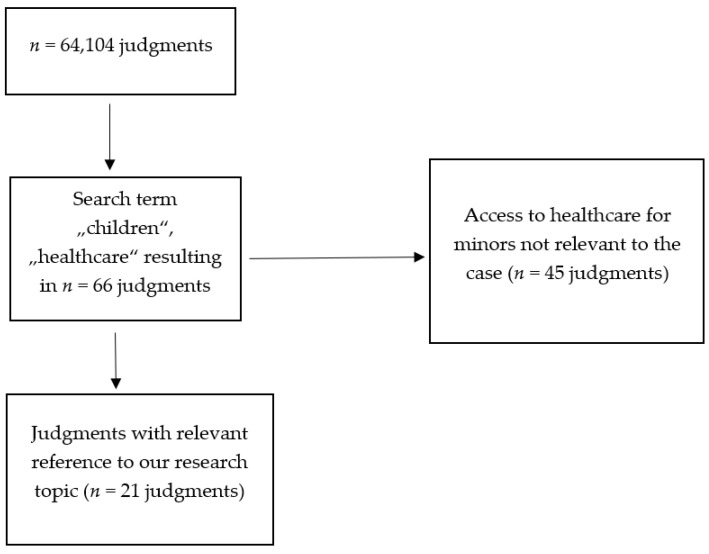
Flowchart of the search (*n* refers to the size of sampled judgments).

In our study, we analyzed judgments of the ECtHR, which were retrieved from the database HUDOC on 30 April 2021, using the search terms “child”, and “healthcare”. HUDOC is a collection of the case-law and judgments of the ECtHR. For the purpose of this analysis, we used the standard setting of the HUDOC’s search engine, providing access to judgments by the Chamber and the Grand Chamber, accessible under https://hudoc.echr.coe.int/eng#{%22documentcollectionid2%22:[%22GRANDCHAMBER%22,%22CHAMBER%22]} (accessed on 30 April 2021). This search yielded *n* = 66 results, which were all read. Only judgments that clearly referred to our research topic were included in further steps of our analysis. Excluded were *n* = 45 judgments that did not concurrently refer to minors and access to healthcare as well as cases in which the Court did not rule upon the violation of minors’ right to access healthcare (Figure 1).

### 2.1. Descriptive Statistics

Descriptive Statistics were performed on the Articles of the Convention, which were involved in judgments under investigation. The ECtHR often ruled on several Articles in one case and even on different paragraphs or aspects of one single Article. For example, it held that the substantive aspect of Article 3 was violated while the procedural aspect was not. We counted a judgment as involving an Article of the Convention if the ECtHR found the Article admissible and ruled on it. To this, we also included judgments with clear reference to the Convention on the Rights of the Child, which are of particular interest to our research topic. A violation was counted if a judgment contained at least one violation of the aforementioned Articles of the European Convention on Human Rights and the Convention for the Rights of the Child, even if the ECtHR held that other aspects of these Articles were not violated in the same case.

### 2.2. Thematic Analysis

A thematic analysis was performed on judgments adhering to the inclusion criteria [15]. Judgments were explored for recurring themes with a focus on the interplay between minors and their right to non-discriminatory access to healthcare. Thematic categories were formed inductively during this process and were critically discussed in the multi-professional team of authors, including physicians (F-A.T. and F.S.), an expert in the history, philosophy and ethics of medicine (F.S.), a mathematician (M.N.) and a political scientist (M.O.) to reduce personal or professional bias. These thematic categories were formulated on the basis of both prevalence of the themes in the research sample and because they capture important statements in relation to the research question. The themes were therefore not deduced from already existing literature on our research topic and are supposed to represent thematic patterns within judgments, which do not depend on exclusively quantifiable measures. This qualitative approach is acknowledged in the methodological literature on thematic analysis [16]. Each category is illustrated by one or more case reports. These themes do not necessarily constitute the core aspects of the Court’s deliberations; however, they provide an important illustration of situations, in which minors faced barriers in access to healthcare.

## 3. Results

From *n* = 64,104 judgments archived online in the HUDOC database, the search term, child’ and ‘healthcare‘ yielded *n* = 66 results. Access to healthcare for minors played a role in *n* = 21 judgments, which are listed in Table 1. As categories are not mutually exclusive, some cases (*n = 5*) have been grouped into several categories. Two cases included two judgments by different chambers of the ECtHR. The analyzed 21 cases cover the time period between years 2012 and 2021. All *n* = 45 judgments excluded from our analysis did not concurrently address the topic of minors and access to healthcare.

### 3.1. Countries

The *n* = 21 judgments involved applications against *n* = 13 countries. Among those countries, *n* = 10 were high-income economies according to the World Bank. Applications were filed against Russia (*n* = 4), The Czech Republic (*n* = 3), Bulgaria, Poland and Germany (each *n* = 2), France, Spain, Lithuania, Slovakia, Slovenia, Turkey, Hungary and Cyprus (each *n* = 1).

### 3.2. Articles of the European Convention on Human Rights

The Convention does not explicitly refer to the right to access to healthcare. However, discriminations in accessing healthcare were stated by the ECtHR in the context of violations of other Articles of the Convention. Table 2 gives an overview of all Articles, which were involved in judgments under investigation. 38.09% of judgments (*n* = 8) involved violations of Article 8 (Right to respect for private and family life), 33.32% of judgments (*n* = 7) involved violations of Article 3 (prohibition of torture), 23.8% of judgments (*n* = 5) involved violations of Article 5 (Right to liberty and security), 14.28% of judgments (*n* = 3) involved violations of Article 13 (Right to an effective remedy) and 4.76% of judgments (*n* = 1) involved violation of Article 2 (Right to life).

### 3.3. Articles of the Convention on the Rights of the Child

Table 3 contains the summary of all Articles of the Convention on the Rights of the Child, which were involved in judgments under investigation. 28.57% of judgments (*n* = 6) involved Article 3 (Best interests of the child), 23.8% of judgments (*n* = 5) involved Article 20 (Protection of children without families), 14.28% of judgments (*n* = 3) involved Article 37 (Rights of the child during detention), 9.52% of judgments (*n* = 2) involved Article 9 (Keeping families together) and Article 24 (Provision of healthcare and safe environment).

### 3.4. Categories

We formed 5, partially overlapping categories, which represent recurring themes regarding our research topic. These themes are: governance failure (*n* = 11), status of refugee, asylum seeker or migrant (*n* = 5), parental home (*n* = 5), maternity and birth (*n* = 4) and others (*n =* 2).

### 3.5. Governance Failure

Governmental failures of healthcare provision extended to discrimination (i) in public institutions (detention zones, refugee camps, orphanages) and (ii) on grounds of deficient legislation in the Member States.

(i): Minors in public structures, such as orphanages can be especially susceptible to discrimination and mistreatment. In the case of X. and others v. Bulgaria (22457/16), two minors reported sexual abuse in a public orphanage in Bulgaria and sought a decision of the Court in reference to Article 3 and 8 of the Convention. The Court stated that the Bulgarian authorities failed to exceed all necessary resources of medical assessments of the alleged sexual abuse of orphans (§ 219).

(ii): In the case P. and S. v. Poland (57375/08), the applicants, a mother with her minor daughter, sued against the detention of the minor applicant after having sought an abortion. The minor applicant became a victim of sexual abuse and tried to interrupt the consequent pregnancy. Instead of accessing adequate gynecological treatment, she was put into detention for “educational supervision” by the local authorities, who feared that the minor applicant could have had illegal “recourse to abortion” (§ 148). In its judgment, the Court referred to the high vulnerability of the minor applicant (§ 162, 164, 166) and noticed inter alia a violation of Article 8 (Right to respect for private and family life) of the Convention as regards the determination of access to lawful abortion.

### 3.6. Status of Refugee, Asylum Seeker or Migrant

In this category, we distinguished two groups: (i) minors with a status of refugees or asylum seekers and (ii) migrants.

(i): In the case of Khan v. France (12267/16) the applicant, an 11-year-old boy from Afghanistan, settled at a heath near the Calais refugee camp. Having already reported physical abuse during his journey to Europe, his subsequent situation in the Calais area was characterized by „very poor conditions of hygiene due to inadequate sanitation, drainage and waste collection facilities, and […] limited access to safe drinking water and healthcare“ (§ 81). In consequence, the applicant complained, that the French Republic did not follow its duties to provide adequate protection for minor refugees in connection to Article 3. In its judgment, the Court referred inter alia to Article 3 of the CRC, which demands “the care or protection of children […] in the areas of safety [and] health” (CRC, Article 3).

(ii): In the case of R.R. and others v. Hungary (36037/17) the applicants, an Iranian-Afghanian family with three minor children, were held in the transit zone near the border of Hungary and Serbia. Sanitary conditions and provision of healthy nutrition were dramatically insufficient and their status as refugees or persons in need was initially not confirmed by the Hungarian officials. The applicants submitted that, although requested, the youngest applicant child had not been given the vaccines “recommended at six months” (§ 18). The Court stated that Article 3 (prohibition of torture) of the Convention has been violated and referred in its judgment to Article 22 (Rights of a child seeking refugee status) of the CRC, which demands humanitarian protection of minors seeking refugee status by States Parties. Another case in this category, G.B. and Others v. Turkey (4633/15), contained the legal action of a family from Russia, who immigrated to Turkey following religious and political persecution. After having tried to cross the Turkish-Syrian border, the applicants, a mother together with her three minor children, were placed in a Turkish detention center. The applicant mother reported “unsuitable” conditions for the “mental and physical health” of her children (§ 27). Additionally, her “oral and written requests for the provision of the three children’s basic needs, including their requests for urgent medical care, had been ignored by the authorities”. Regarding the situation of the minor applicants, the Court referred in its judgment to Article 3 of the CRC and held inter alia that Article 3 of the Convention (prohibition of torture) was violated on account of the conditions of the applicants‘ detention.

### 3.7. Parental Home

This category extended to minors, which were deprived of adequate access to healthcare due to their living conditions in their parental homes. All cases contained removals of minors from their parents by public authorities because parents could not fulfill basic requirements for the life and health of their children.

In Tlapak and Others v. Germany (11308/16 and 11344/16) the applicants, parents and their three minor children, were parts of a religious community (Twelve tribes). Footage, which was presented to the German Court, proved the use of violent education measures like corporal punishments on children in the community, which finally led to the compulsive removal of all children to a foster family. Both adult applicants were continuously convinced that those practices were appropriate and necessary parenting methods and rejected in this context any kinds of therapeutical interventions, e.g., their “disagreement about the son attending a state school and play therapy” (§ 27). In its judgment, the court referred to Article 3 § 1 (Best interest of the child regarding his/her right to health) of the CRC and held no violation of Article 8 (Right to respect for private and family life) of the Convention. The Court differentiated between the best interests of the child and the interests of parents, which following Article 8 could be overridden by the interests of the child in cases of doubts (§ 81).

### 3.8. Maternity and Birth

During the process of birth and the postnatal period, minors were proven to be deprived of adequate healthcare in *n* = 4 cases. These cases referred to the right of mothers to give birth under secure and appropriate conditions. Due to the specific physical interdependence between mothers and their children, violations of human rights of the mother also affect the right of newborn minors to receive adequate medical treatment.

In Dubská and Krejzová v. The Czech Republic (28859/11, 28473/12) the applicants were two mothers who had to give birth in public hospitals, although both of them repeatedly called for being assisted by a midwife during a home birth. It is important to state that although the Grand Chamber of the Court did not find a breach of the Convention, it retook its formerly expressed opinion that “birth, and in particular the circumstances in which a child is born, forms part of a child’s, and subsequently the adult’s, private life guaranteed by Article 8 of the Convention” (§ 162). This case constituted an important reference for further decisions of the Court. For example, in the case of Kosaité-Čypiené and others v. Lithuania (69489/12), referring to the case Dubská and Krejzová v. The Czech Republic (28859/11, 28473/12), the Court made a provision that “in no circumstances should a child be deprived of his or her right of access to healthcare services on the grounds that he or she was born outside of a medical facility. The best interests of the child must be a primary consideration in all actions concerning children, whether undertaken by public or private social-welfare institutions” (§ 109).

### 3.9. Other Cases

In this category, we included cases, in which the ECtHR’s judgment did not refer directly to the assessment of discrimination of minor applicants. Nevertheless, in some cases, we detected aspects, which are linked to our research topic and should therefore not be dismissed. For example, in the case of V.C. v. Slovakia (18968/07), a mother of Roma ethnic origin experienced degrading treatment during her birth delivery, which finally resulted in her involuntary sterilization. The general conditions of both, mother and child, were highly hostile during their stay at the hospital, which could have therefore also affected the health of the newborn child.

## 4. Discussion

The research was conducted to study the situation of access modalities of minors in the healthcare system. The results of our analysis show that the question of access to healthcare for minors includes a relatively small number of the judgments of ECtHR (*n* = 21). This can be caused by a low number of applications to national courts, especially in situations of sexual abuse of children, violence, and cases of minor migrants and refugees. Trauma, shame and lack of legal representation for children precludes such cases from being detected and legally considered. As the applications to the ECtHR first need to undergo national legal remedies, these cases are fittingly considered as a “tip of an iceberg”.

Nevertheless, our analysis of ECtHR judgments reveals an important intersection of medicoehtical and legal questions of access to healthcare, human dignity, and justice. It has been demonstrated that minors frequently face barriers when accessing health services [9,17,18]. Our analysis shows that access to healthcare for minors can be constrained in several situations. Governmental failure to guarantee healthcare provision in public institutions can have direct consequences to minors’ health. Deficient national legislation can lead to exclusion of minors from access to particular healthcare services. Moreover, minor migrants and asylum seekers constitute a vulnerable group, which is particularly exposed to health risks due to their legal status in the host countries, conditions in which they live and limited access to healthcare resources. Parental failure to fulfill requirements for the health of their children can lead to deprivation of access to healthcare. Also, in a specific situation of birth, access to healthcare for minors can be limited, if, due to the decision of the parents, the birth takes place outside a healthcare facility. The judgments of the ECtHR illustrate such situations. It is clear that in its judgments the ECtHR emphasizes the particular vulnerability of children, which require exceptional protection, especially by the Member States. The ECtHR also clearly states that this vulnerability needs to be recognized by national legal structures, as well as healthcare systems and healthcare professionals [19]. In the following, we discuss the findings of the main thematic categories identified in this research.

### 4.1. Governance Failure

Sexual abuse of minors constitutes a serious threat to their somatic and mental health. Prevalence rates of this phenomenon are difficult to assess due to differences in the character of the offense and methods of survey [20]. A study of the male population group in Finland showed that 0.3% of the surveyed had sexual contact with a person under the age of 16 [21]. The risk of child sexual abuse increases in cases of out-of-home youth, i.e., children living in a state institution, kept in detention or living on the street. The negative health outcomes of sexual abuse for children vary from severe psychiatric sequelae to somatic outcomes. Affected children are at increased risk of post-traumatic stress disorder (PTSD), depression, anxiety and inappropriate sexual behavior throughout their life span [22,23,24]. Survivors of child abuse are prone to sexual revictimization in adolescence and adulthood [25]. These mental states can influence further behavior of the victims, leading to alcoholism and drug abuse or suicide attempts, which subsequently have a cumulative negative effect on somatic diseases such as heart, lung and liver diseases or cancer [26]. Therefore, child protection strategies implemented by the government are of special importance, as they enable the quick detection of child abuse and the provision of quick access to therapeutic treatments, such as trauma-focused cognitive-behavioral therapy, which has been demonstrated as an effective treatment for the psychological sequelae of sexual abuse cases [27]. In its judgments, the ECtHR reiterates that States have an obligation to establish a legislator and regulatory framework to protect the physical and psychological integrity of individuals (X. and others v. Bulgaria, 22457/16, § 179, ECHR, 1999-II). This obligation should be applied in cases of rape or sexual abuse of children. Here, the context of protection of health and well-being of children as a particularly vulnerable group gains importance, as substantiated in Articles 3 and 19 of the CRC. Especially children deprived of parental support and under the care of public institutions are in a predominantly vulnerable situation. Breach of such obligations can constitute a potential violation of Article 3 of the Convention prohibiting inhuman or degrading treatment. This includes the question of access to pregnancy termination as illustrated in the case P. and S. v. Poland. A State’s obligation in case of pregnancy termination includes the need to adopt detailed guidelines for healthcare practitioners to prevent suffering for the victim, minimize the trauma and promote the healing (P. and S. v. Poland, 57375/08, 3, ECHR, 1999-II).

### 4.2. Status of Refugees, Asylum Seekers or Migrants

Unaccompanied or accompanied minor migrants constitute a significant number of displaced persons worldwide. In Europe in 2015, the number of minor migrants, asylum seekers, and refugees exceeded 300,000, of which more than 90,000 were unaccompanied [28]. Due to lack of health information, language obstacles and cultural differences, minor migrants and refugees experience barriers to adequate, timely and appropriate healthcare [9,17,18,29]. Migrating children are susceptible to communicable diseases because of physical stress, stays in overcrowded means of transport or refugee centers, and limited opportunities for personal space, hygiene and healthcare [30]. Therefore, special attention should be put to their vaccination needs. Moreover, considering their mental health, unaccompanied refugee minors have a disproportionately high prevalence of psychiatric morbidities [31]. This is in many cases caused by their experience of trauma in the countries of origin and during the travel to the destination country. Such circumstances increase the vulnerability of minors and can manifest in health-seeking behavior [32]. The ECtHR observes that extreme and inhumane living conditions can contribute to decline in the somatic and mental state of migrants, including outbreak or aggravation of diseases, conditions of anxiety depression and impaired mental health. Therefore, the ECtHR holds the position that minors, irrespective of the status of their refugee application, fall into the category of the most vulnerable individuals in the society (Khan v. France, 12267/16, § 74, ECHR, 1999-II). The States, in which minor migrants stay, have a particular obligation to guarantee suitable conditions of shelter, access to appropriate healthcare, schooling or training. Provision of adequate healthcare, according to the ECtHR has its fundament in international law, particularly in Article 3 and Article 22 of the Convention on the Rights of the Child and Article 19 of the European Union Directive 2013/33/EU. According to the ECtHR, the failure of a State to provide such conditions and protection of a minor constitutes a breach of human rights and violation of Article 3 of the Convention, prohibiting inhumane or degrading treatment.

### 4.3. Parental Home

According to the World Health Organization (WHO), child maltreatment is the physical, sexual and/or emotional abuse and/or neglect of children under 18 years of age [33]. In their report on preventing child maltreatment from 2013, the WHO considers child abuse and neglect not only as a matter for criminal justice or a social issue, but also a concern for public health [34]. Child victimization, which covers various forms of violence, neglect and mistreatment, has a major negative impact on concurrent and subsequent psychopathology, health morbidity, and compromised development [35]. Especially family violence is a risk factor for child victimization [36]. Therefore, the WHO demands from the national health ministries to take a leadership role in ensuring that national policies and plans for preventing child maltreatment [34]. With its ruling, the ECtHR states that the best interest of the child regarding his/her right to health, manifested in Article 3 § 1 of the CRC, demands special protection when the parental home does not provide the necessary care and attention and that this value may even overrule the right to respect for private and family life, stated in Article 8 of the Convention. In cases of allocating parental decision-making capacities to state institutions, the Courts position nevertheless tended clearly towards prioritizing the responsibilities of parents [14] (pp. 191–192). Additionally, questions related to compulsory vaccinations or other medical interventions of minors were also repeatedly subject to the decisions of the Court and will in future need to be filled out by further judgments [14] (p. 197).

### 4.4. Maternity and Birth

The ECtHR recognizes that States Parties must ensure appropriate access to “health services in connection with pregnancy […] and the post-natal period” [19]. In our cases under investigation, we dealt with the right of mothers to give birth at home and the best interest of newborn children. Although hospitals are the safest setting for birth [37,38], the right of a woman to make a medically informed decision about the form of delivery should be respected by healthcare professionals for the sake of the mother and her newborn child [39,40]. Implementation of policy to support planned home birth, including the policy that endorses building or sustaining a home birth infrastructure in parallel to the efforts to build capacity for facility-based birth should be encouraged [41]. It should be noticed, that although not represented in judgments of the ECtHR, this category reaches also out to challenges regarding perinatal access to healthcare in more life-threatening contexts like the context of flight [42]. In its rulings, the ECtHR focuses on autonomous decision-making of mothers, taking under account the circumstances of the birth and balancing it with the principles of beneficence and non-maleficence both for the mother and the child.

### 4.5. Limitations

Our categorization of *n* = 21 judgments does not reflect the whole group of minor patients and the violation of their human rights when entering healthcare systems. Some subgroups of minors might be underrepresented in our analysis as their right to sue for non-discriminatory access to healthcare significantly depends on their ability to access justice. As was already pointed out, our analysis points towards a mere tip of an iceberg and includes examples of human rights violations for minor patients that were considered by the ECtHR.

## 5. Conclusions

Our analysis of ECtHR judgments revealed that minors are subject to violations of their human rights on different levels. In the context of access to healthcare, their condition is already characterized by a vulnerability in facing discrimination and therefore bears the risk of “double marginalization”—first as children and second as, e.g., migrant—with adverse outcomes for health status resulting from both conditions. Although violations of children rights noticed by ECtHR have thoroughly been studied, they are rarely presented in the context of healthcare [14] (p. 197). The results of our research therefore augment the range of the possible circumstances under which discrimination in access to healthcare for minors has already been described in current literature. Our study is primarily directed to healthcare professionals in pediatrics who need awareness of the social, legal and ethical circumstances of the patients’ situation, especially in cases with increased risks of marginalization. Healthcare professionals are often at the forefront of encountering structural discrimination in the healthcare system and therefore need to be sensitized for discrimination mechanisms from the ethical point of view. Secondly, governments and policymakers should ensure non-discriminatory access to healthcare in their national contexts with particular emphasis on patients with vulnerability profiles. Our research revealed that in this context particular attention must be paid to public institutions like orphanages or refugee camps. Thirdly, legislation must be written in a manner that reflects the difficulties of discriminated groups in healthcare systems and therefore effectively tackles all challenges of their condition.

## Figures and Tables

**Table 1:** Judgments in which access to healthcare for minors played a role (*n* = 21).

Category	Case	Violation of Articles of the Convention	Aspects of Judgments Related to Access to Healthcare for Minors
Governance failure	G.B. and Others v. Turkey (4633/15)	yes (Articles 3, 5, 13)	access to urgent medical care and healthy living conditions
	P. and S. v. Poland (57375/08)	yes (Articles 3, 5, 8)	access to lawful abortion
	X and Others v. Bulgaria (22457/16) *	yes (Procedural limb of Article 3)	access to healthcare in orphanage
	Dubská and Krejzová v. The Czech Republic (28859/11 28473/12) *	no	access to healthcare during birthing at home
	Khan v. France (12267/16)	yes (Article 3)	access to healthcare and sanitation in refugee camp
	R.R. and Others v. Hungary (36037/17)	yes (Articles 3, 5)	access to vaccination for minor refugees
	Kurić and Others v. Slovenia (26828/06)	yes (Articles 8, 13)	access to healthcare after loss of citizenship
	Bistieva and Others v. Poland (75157/14)	yes (Article 8)	access to healthy living conditions in a detention center for migrants
	Blyudik v. Russia (46401/08)	yes (Articles 5, 8)	access to medical examination
Refugee, asylum or migration	R.R. and Others v. Hungary (36037/17)	yes (Articles 3, 5)	access to vaccination of minor refugees
	G.B. and Others v. Turkey (4633/15)	yes (Articles 3, 5, 13)	access to urgent medical care and healthy living conditions
	Khan v. France (12267/16)	yes (Article 3)	access to healthcare and sanitation in refugee camp
	M.A. v. Cyprus (41872/10)	yes (Articles 2, 3, 5, 13)	access to healthcare for minor asylum seekers
	Bistieva and Others v. Poland (75157/14)	yes (Article 8)	access to healthy living conditions in a detention center for migrants
Parental home	Vavřička and Others v. The Czech Republic (47621/13 3867/14 73094/14 19298/15 19306/15 43883/15)	no	access to vaccination
	Tlapak and Others v. Germany (11308/16 11344/16)	no	access to psychotherapy
	Wetjen and Others v. Germany (68125/14 72204/14)	no	access to healthcare
	V.D. and Others v. Russia (72931/10)	yes (Article 8)	healthy living conditions in families
	Ageyev v. Russia (7075/10)	yes (Article 8)	access to healthcare against the will of parents
Maternity and birth	Konovalova v. Russia (37873/04)	yes (Article 8)	access to adequate healthcare and privacy during birthing
	Dubská and Krejzová v. The Czech Republic (28859/11 28473/12) *	no	access to healthcare during birthing at home
	Kosaité-Čypiené and Others v. Lithuania (69489/12)	no	access to healthcare during birthing at home
Others	V.C. v. Slovakia (18968/07)	yes (Articles 3, 8)	hostile perinatal treatment conditions towards a mother and her newborn child
	N.D. and N.T. v. Spain (8675/15 8697/15)	no	medical assistance following illegal migration

* Cases which included two judgments by different chambers of the ECtHR.

**Table 2:** Frequencies of Articles of the European Convention on Human Rights involved in the *n* = 21 judgments under investigation.

Articles of the European Convention on Human Rights	Judgments Involving This Article	Judgments in Which at Least One Violation of This Article Was Found
Article 2	*n* = 1 (4.76%)	*n* = 1 (4.76%)
Article 3	*n* = 8 (38.09%)	*n* = 7 (33.32%)
Article 5	*n* = 5 (23.8%)	*n* = 5 (23.8%)
Article 8	*n* = 15 (71.42%)	*n* = 8 (38.09%)
Article 13	*n* = 5 (23.8%)	*n* = 3 (14.28%)
Article 34	*n* = 2 (9.52%)	*n* = 0
Article 35	*n* = 6 (28.57%)	*n* = 0
Article 41	*n* = 4 (19.04%)	*n* = 0
Article 4 of Protocol No 4	*n* = 2 (9.52%)	*n* = 0

**Table 3:** Frequencies of Articles of the UN Convention on the Rights of the Child involved in the *n* = 21 judgments under investigation.

Articles of the Convention on the Rights of the Child	Judgments Involving This Article
Article 2	*n* = 1 (4.76%)
Article 3	*n* = 6 (28.57%)
Article 9	*n* = 2 (9.52%)
Article 19	*n* = 1 (4.76%)
Article 20	*n* = 5 (23.8%)
Article 22	*n* = 1 (4.76%)
Article 24	*n* = 2 (9.52%)
Article 37	*n* = 3 (14.28%)
Article 44	*n* = 1 (4.76%)

## Data Availability

Publicly available datasets were analyzed in this study. This data can be found here: https://hudoc.echr.coe.int/eng#{%22documentcollectionid2%22:[%22GRANDCHAMBER%22,%22CHAMBER%22]} (accessed on 10 September 2021).
